# Regulation of intestinal epithelial cells transcriptome by enteric glial cells: impact on intestinal epithelial barrier functions

**DOI:** 10.1186/1471-2164-10-507

**Published:** 2009-11-02

**Authors:** Laurianne Van Landeghem, Maxime M Mahé, Raluca Teusan, Jean Léger, Isabelle Guisle, Rémi Houlgatte, Michel Neunlist

**Affiliations:** 1 INSERM, U913, Nantes, F-44000 France; 2 Université de Nantes, Faculté de Médecine, Nantes, F-44000 France; 3 CHU Nantes, Institut des Maladies de l'Appareil Digestif, Nantes, F-44000 France; 4 INSERM, U915, Institut du Thorax, Nantes, F-44000 France; 5 CHU Nantes, Institut du Thorax, Nantes, F-44000 France

## Abstract

**Background:**

Emerging evidences suggest that enteric glial cells (EGC), a major constituent of the enteric nervous system (ENS), are key regulators of intestinal epithelial barrier (IEB) functions. Indeed EGC inhibit intestinal epithelial cells (IEC) proliferation and increase IEB paracellular permeability. However, the role of EGC on other important barrier functions and the signalling pathways involved in their effects are currently unknown. To achieve this goal, we aimed at identifying the impact of EGC upon IEC transcriptome by performing microarray studies.

**Results:**

EGC induced significant changes in gene expression profiling of proliferating IEC after 24 hours of co-culture. 116 genes were identified as differentially expressed (70 up-regulated and 46 down-regulated) in IEC cultured with EGC compared to IEC cultured alone. By performing functional analysis of the 116 identified genes using Ingenuity Pathway Analysis, we showed that EGC induced a significant regulation of genes favoring both cell-to-cell and cell-to-matrix adhesion as well as cell differentiation. Consistently, functional studies showed that EGC induced a significant increase in cell adhesion. EGC also regulated genes involved in cell motility towards an enhancement of cell motility. In addition, EGC profoundly modulated expression of genes involved in cell proliferation and cell survival, although no clear functional trend could be identified. Finally, important genes involved in lipid and protein metabolism of epithelial cells were shown to be differentially regulated by EGC.

**Conclusion:**

This study reinforces the emerging concept that EGC have major protective effects upon the IEB. EGC have a profound impact upon IEC transcriptome and induce a shift in IEC phenotype towards increased cell adhesion and cell differentiation. This concept needs to be further validated under both physiological and pathophysiological conditions.

## Background

The intestinal epithelial barrier (IEB) is the first boundary between the organism and the luminal environment. It plays a dual role by allowing the passage of nutrients and electrolytes but preventing the passage of pathogens. The maintenance of its homeostasis is of utmost importance for the survival of the organism. The IEB is formed by a monolayer of specialized intestinal epithelial cells (IEC) under constant renewal and maintained together *via *various cell-to-cell and cell-to-matrix interactions. The IEB is part of a complex network of specialized cell types constituting its microenvironment such as immune cells, subepithelial fibroblasts, endothelial cells or luminal bacteria. Emerging evidences suggest that under physiological conditions, the IEB's functions are actively regulated by its cellular microenvironment [1-3]. For instance, myofibroblasts have been shown to enhance epithelial cell proliferation and intestinal epithelial restitution [4]. In addition, microbiota have been shown to control both the maturation and the maintenance of the IEB [5].

The enteric nervous system (ENS) is also a major constituent of the cellular microenvironment of the IEB. Indeed IEB and, in particular, the proliferative compartment of the crypts are densely innervated by nerve fibres originating mainly from the submucosal plexus. Recent data have shown that, besides controlling secretory processes, activation of enteric neurons can reduce IEC proliferation and barrier permeability, in particular *via *the release of vasoactive intestinal peptide (VIP) [6-8]. Enteric neurons innervating the IEB are also closely associated with enteric glial cells (EGC), the major constituent of the ENS.

For many years, EGC have been considered as mainly passive and structural cells supporting neurons and ganglions. However, this concept has lately been revisited mainly focused on the role played by astrocytes in the central nervous system (CNS) [9-11]. Besides controlling and regulating neuronal functions, increasing evidence suggests that EGC could be major regulators of IEB functions, similar to astrocytes controlling blood brain barrier functions [10]. Supporting this concept, recent data have demonstrated that EGC can profoundly inhibit IEC proliferation, in part *via *the liberation of TGF-β1 [12]. EGC also decrease IEB paracellular permeability *via *the release of S-nitrosoglutathione (GSNO) [13]. Furthermore, *in vivo *lesions of EGC network increase IEB paracellular permeability and IEC proliferation and, at term, lead to major lethal intestinal inflammation [13-15]. However, the role of EGC in the control of other major IEC functions such as cell differentiation, cell-to-cell or cell-to-matrix adhesion, and the associated regulatory pathways remains largely unknown.

Therefore, in our study, we combined transcriptomic studies as well as functional studies to determine the impact of EGC on the regulation of major genes and functions involved in IEB homeostasis. Microarray approach was used to identify EGC-induced modifications in gene expression profiling of proliferating Caco-2. The identified genes and related functional pathways are consistent with the concept that EGC are a major constituent of the IEB microenvironment favoring barrier protection.

## Results and Discussion

### Enteric glial cells modulate intestinal epithelial cells transcriptome

#### Microarray experiments

We performed microarray analysis of EGC influence on the transcriptome of Caco-2 cells using oligonucleotide chips (Cancerochips) developed at West Genopole transcriptome core facility of Nantes. These microarrays contain around 6,864 genes and are dedicated to gene expression studies in Caco-2 cell line as well as to gene expression signature studies of multiple tumors. Caco-2 cells were cultured onto Transwell filters in the absence or presence of EGC seeded at the bottom of the wells for 8 or 24 hours. The Transwell filters did not allowed any contact between IEC and EGC, thus implicating only paracrine communication between the two cell types.

**Figure 1:**
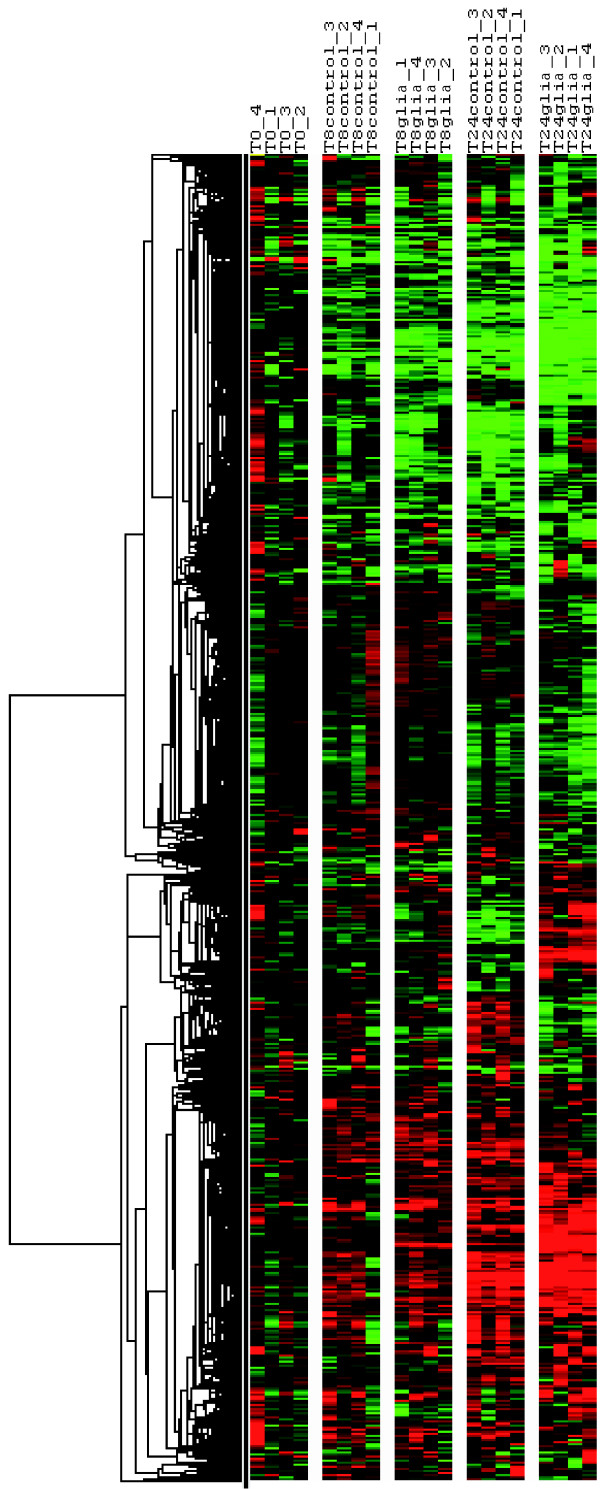
**Hierarchical clustering of expression data**. Four individual microarrays were used per condition. Hierarchical clustering was performed on genes using Gene Cluster. Each ratio was normalized to the median of the t = 0 hour-condition values of the corresponding gene. Each column represents an individual array (T0: t = 0 hour condition samples; T8control: t = 8 hours of culture without EGC; T8glia: t = 8 hours of culture in presence of EGC; T24control: t = 24 hours of culture without EGC; T24glia: t = 24 hours of culture in presence of EGC). Each line represents one individual gene. The clustering shows the impact of the time of culture on gene expression profiling in Caco-2 cells. The EGC-induced modulation of IEC transcriptome is highly visible at t = 24 hours.

Hierarchical clustering of the whole data showed the impact of the time of culture as well as the impact of the presence of EGC on the transcriptional profiling of IEC, i.e. Caco-2 cells (Figure 1). We observed changes in IEC transcriptome over the 24 hours of culture in control condition. At 8 hours, differences in transcriptome profiling already existed in control condition as compared to t = 0. In general, the observed changes in differentially expressed genes between t = 0 and t = 8 hours in control conditions were increased in the same way of regulation when reaching t = 24 hours (Figure 1). These changes might be due to the growth and differentiation of the proliferating IEC over the 24 hours of culture. We observed no major differences in gene expression profiling between IEC cultured alone and IEC cultured in presence of EGC at 8 hours of culture. In contrast, at 24 hours, EGC presence led to consistent and major changes in IEC gene expression profiling (Figure 1).

#### Gene expression modulated by EGC

**Figure 2:**
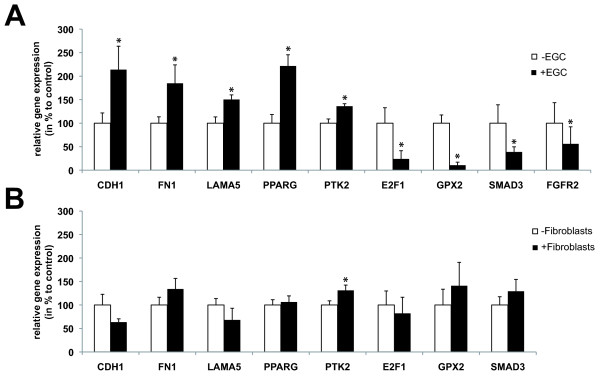
**Enteric glial cells EGC) and fibroblasts differentially modulated intestinal epithelial cell (IEC) transcriptome**. (A). Real-time quantitative PCR studies on *CDH1*(n = 5), *FN1 *(n = 7), *LAMA5 *(n = 6), *PPARG *(n = 5), *PTK2 *(n = 5), *E2F1 *(n = 7), *FGFR2 *(n = 6), *GPX2 *(n = 8), *SMAD3 *(n = 7) gene expression in IEC cultured for 24 hours alone (- EGC) or in presence of EGC(+ EGC) confirmed that EGC significantly modulate the level of expression of genes identified by the microarrays data analysis as differentially expressed in IEC cultured in presence of EGC (*p < 0.05; Mann-Whitney test). (B). In contrast, real-time quantitative PCR studies on *CDH1 *(n = 5), *FN1 *(n = 5), *LAMA5 *(n = 5), *PPARG *(n = 5), *PTK2 *(n = 5), *E2F1 *(n = 5), *GPX2 *(n = 5), *SMAD3 *(n = 5) gene expression in IEC cultured for 24 hours alone (- fibroblasts) or in presence of fibroblasts (+fibroblasts) showed a differential regulation of gene expression as compared to EGC effects (*p < 0.05; Mann-Whitney test).

Using Genespring software, we aimed to identify statistically significant differences in gene expression profiling between Caco-2 cells cultured alone or in presence of EGC. After 8 h of culture, no significant difference in gene expression profiling was found between IEC cultured alone (control condition) or in presence of EGC ("glia" condition). However, after 24 hours of culture, we identified 116 genes differentially expressed between control and EGC conditions by using two different strategies. Benjamini and Hochberg False Discovery Rate method was used to determine 98 differentially expressed genes between control and glia conditions at t = 24 hours, and we also selected 27 genes with a two-fold change and Student's t-test p-value less than or equal to 0.05. Among the 116 differentially expressed genes, 46 genes were down-regulated and 70 were up-regulated in IEC cultured with EGC as compared to control (Table 1, 2). Quantitative PCR was also performed on various genes to validate the microarray results. In particular, results showed an EGC-induced increase of *CDH1, FN1*, *LAMA5*, *PPARG, PTK2 *mRNA expression in IEC and a decrease of *E2F1, FGFR2, GPX2 *and *SMAD3 *mRNA expression in IEC, similar to the data obtained with microarrays (Figure 2A). We next sought to determine the specificity of EGC effects upon IEC transcriptome by characterizing the impact of fibroblasts on the expression of these genes in IEC. Under identical culture conditions, we showed that fibroblasts increased expression of *PTK2 *but did not significantly modify gene expression of *CDH1, FN1*, *LAMA5*, *PPARG, E2F1, GPX2 and SMAD3 *in IEC (Figure 2B).

**Table 1:** List of the genes up-regulated by enteric glial cells in intestinal epithelial cells.

Gene Symbol	Genbank	Description	% up-regulation (/control)	Fold difference
TXNIP	NM_006472	thioredoxin interacting protein	217,60	3,18
ANKRD1	NM_014391	ankyrin repeat domain 1 (cardiac muscle)	169,22	2,69
FN1	U42593	fibronectin 1	152,16	2,52
TUBB3	NM_006086	tubulin, beta 3	149,94	2,50
MGLL	AJ270950	monoglyceride lipase	135,97	2,36
METTL7A	NM_014033	methyltransferase like 7A	132,96	2,33
PKN2	NM_006256	protein kinase N2	128,33	2,28
/	XM_166201	synonyms: KIAA0056, MGC104671; Homo sapiens KIAA0056 protein (hCAP-D3), mRNA.	115,09	2,15
EPB41L2	NM_001431	erythrocyte membrane protein band 4.1-like 2	110,50	2,11
AASS	AJ007714	aminoadipate-semialdehyde synthase	110,15	2,10
ACTG2	NM_001615	actin, gamma 2, smooth muscle, enteric	102,56	2,03
B4GALT5	NM_004776	UDP-Gal:betaGlcNAc beta 1,4- galactosyltransferase, polypeptide 5	102,38	2,02
SNX2	NM_003100	sorting nexin 2	102,38	2,02
VIP	NM_003381	vasoactive intestinal peptide	102,33	2,02
EIF4A2	NM_001967	eukaryotic translation initiation factor 4A, isoform 2	102,14	2,02
/	NM_019027	RNA-binding protein	101,66	2,02
POLR3F	NM_006466	polymerase (RNA) III (DNA directed) polypeptide F, 39 kDa	94,46	1,94
PNRC1	NM_006813	proline-rich nuclear receptor coactivator 1	93,24	1,93
NPPB	NM_002521	natriuretic peptide precursor B	83,97	1,84
/	BC017857	Homo sapiens cDNA clone IMAGE:4690793, with apparent retained intron.	82,20	1,82
KRT8	NM_002273	keratin 8	81,96	1,82
SAT1	NM_002970	spermidine/spermine N1-acetyltransferase 1	80,24	1,80
ASS1	NM_000050	argininosuccinate synthetase 1	76,73	1,77
S100A11P	NM_021039	/	75,22	1,75
SLC7A7	NM_003982	solute carrier family 7 (cationic amino acid transporter, y+ system), member 7	72,31	1,72
HAGH	NM_005326	hydroxyacylglutathione hydrolase	69,54	1,70
BNIP3L	AF536326	BCL2/adenovirus E1B 19 kDa interacting protein 3-like	69,19	1,69
/	AF195968	PRR5-ARHGAP8 fusion	68,01	1,68
BNIP3	NM_004052	BCL2/adenovirus E1B 19 kDa interacting protein 3	67,84	1,68
IL18	NM_001562	interleukin 18 (interferon-gamma-inducing factor)	67,14	1,67
RDM1	BC038301	RAD52 motif 1	67,11	1,67
FAM107B	NM_031453	family with sequence similarity 107, member B	65,70	1,66
PLAC8	NM_016619	placenta-specific 8	63,77	1,64
SMARCA1	NM_139035	SWI/SNF related, matrix associated, actin dependent regulator of chromatin, subfamily a, member 1	63,72	1,64
PLOD2	NM_000935	procollagen-lysine, 2-oxoglutarate 5-dioxygenase 2	62,87	1,63
TMSB4Y	NM_004202	thymosin, beta 4, Y-linked	62,77	1,63
SCPEP1	NM_021626	serine carboxypeptidase 1	60,96	1,61
LAMA5	NM_005560	laminin, alpha 5	60,53	1,61
LAMC1	NM_002293	laminin, gamma 1 (formerly LAMB2)	59,89	1,60
METAP1	BC030054	methionyl aminopeptidase 1	59,55	1,60
IQGAP2	NM_006633	IQ motif containing GTPase activating protein 2	58,98	1,59
C1orf43	NM_015449	chromosome 1 open reading frame 43	56,86	1,57
CASP4	NM_001225	caspase 4, apoptosis-related cysteine peptidase	55,71	1,56
BTG1	NM_001731	B-cell translocation gene 1, anti-proliferative	54,63	1,55
SLC2A1	K03195	solute carrier family 2 (facilitated glucose transporter), member 1	54,34	1,54
DCTN2	NM_006400	dynactin 2 (p50)	52,68	1,53
TOP2A	NM_001067	topoisomerase (DNA) II alpha 170 kDa	52,42	1,52
KRT18	NM_000224	keratin 18	51,16	1,51
LAMC1	M55210	laminin, gamma 1 (formerly LAMB2)	50,37	1,50
PRC1	BC005140	protein regulator of cytokinesis 1	50,13	1,50
IMPDH2	NM_000884	IMP (inosine monophosphate) dehydrogenase 2	49,46	1,49
/	AF202922	LRP16 protein	48,20	1,48
PLD3	NM_012268	phospholipase D family, member 3	46,99	1,47
RNF4	NM_002938	ring finger protein 4	44,88	1,45
/	AC060225	Homo sapiens 3 BAC RP11-23J16 complete sequence.	42,10	1,42
SMARCA1	NM_003069	SWI/SNF related, matrix associated, actin dependent regulator of chromatin, subfamily a, member 1	41,18	1,41
DYNLT3	NM_006520	dynein, light chain, Tctex-type 3	40,06	1,40
PPARG	NM_015869	peroxisome proliferative activated receptor, gamma	38,92	1,39
GLRX	AF069668	glutaredoxin (thioltransferase)	37,78	1,38
PTK2	NM_153831;NM_005607	PTK2 protein tyrosine kinase 2	37,72	1,38
CDH1	NM_004360	cadherin 1, type 1, E-cadherin (epithelial)	36,87	1,37
RNASE4	NM_002937	ribonuclease, RNase A family, 4	31,40	1,31
CTSH	NM_004390	cathepsin H	29,45	1,29
MKI67	NM_002417	antigen identified by monoclonal antibody Ki-67	29,28	1,29
EIF2A	NM_032025	eukaryotic translation initiation factor 2A, 65 kDa	26,54	1,27
TGFBI	BC000097	transforming growth factor, beta-induced, 68 kDa	25,95	1,26
MLLT3	NM_004529	myeloid/lymphoid or mixed-lineage leukemia (trithorax homolog, Drosophila); translocated to, 3	22,42	1,22
APOBEC3B	NM_004900	apolipoprotein B mRNA editing enzyme, catalytic polypeptide-like 3B	22,20	1,22
ADD3	NM_019903	adducin 3 (gamma)	20,82	1,21
FTH1	NM_002032	ferritin, heavy polypeptide 1	15,97	1,16

**Table 2:** List of the genes down-regulated by enteric glial cells in intestinal epithelial cells.

Gene Symbol	Genbank	Description	% down-regulation (/control)	Fold difference
CARD12	AF376061	caspase recruitment domain family, member 12	83,43	6,04
KLK14	NM_022046	kallikrein 14	62,46	2,66
FGFR2	NM_022970	fibroblast growth factor receptor 2	57,12	2,33
BDP1	NM_018429	B double prime 1, subunit of RNA polymerase III transcription initiation factor IIIB	56,58	2,30
SFRP4	NM_003014	secreted frizzled-related protein 4	55,26	2,24
C6	NM_000065	complement component 6	54,78	2,21
PRKCD	NM_006254	protein kinase C, delta	54,67	2,21
/	XM_066534	Homo sapiens diacylglycerol kinase, kappa (DGKK), mRNA.	52,44	2,10
C20orf133	NM_001033086	chromosome 20 open reading frame 133	52,36	2,10
PRKCQ	NM_006257	protein kinase C, theta	50,50	2,02
CDK5R1	NM_003885	cyclin-dependent kinase 5, regulatory subunit 1 (p35)	50,42	2,02
RPP40	NM_006638	ribonuclease P 40 kDa subunit	47,41	1,90
SLC30A1	AF323590	solute carrier family 30 (zinc transporter), member 1	46,22	1,86
TIMM8A	NM_004085	translocase of inner mitochondrial membrane 8 homolog A (yeast)	41,07	1,70
EBNA1BP2	NM_006824	EBNA1 binding protein 2	36,39	1,57
ITGAE	NM_002208	integrin, alpha E (antigen CD103, human mucosal lymphocyte antigen 1; alpha polypeptide)	36,17	1,57
NOL1	NM_006170	nucleolar protein 1, 120 kDa	33,86	1,51
C6orf66	NM_014165	chromosome 6 open reading frame 66	33,81	1,51
NOL5A	NM_006392	nucleolar protein 5A (56 kDa with KKE/D repeat)	33,30	1,50
BAG1	U46917	BCL2-associated athanogene	32,19	1,47
/	AF123534	nucleolar protein NOP5/NOP58	32,14	1,47
ASAH1	AK025211	N-acylsphingosine amidohydrolase (acid ceramidase) 1	29,98	1,43
TINAGL1	AF236150	tubulointerstitial nephritis antigen-like 1	29,62	1,42
AADAC	NM_001086	arylacetamide deacetylase (esterase)	29,48	1,42
HSPA14	AF112210	heat shock 70 kDa protein 14	29,34	1,42
PSMC6	NM_002806	proteasome (prosome, macropain) 26S subunit, ATPase, 6	29,31	1,41
HNRPDL	D89678	heterogeneous nuclear ribonucleoprotein D-like	28,40	1,40
SAMHD1	NM_015474	SAM domain and HD domain 1	28,12	1,39
TP53RK	NM_033550	TP53 regulating kinase	26,99	1,37
MARK2	NM_004954	MAP/microtubule affinity-regulating kinase 2	26,41	1,36
CCR9	NM_031200	chemokine (C-C motif) receptor 9	24,74	1,33
RGL1	NM_015149	ral guanine nucleotide dissociation stimulator-like 1	24,20	1,32
E2F1	NM_005225	E2F transcription factor 1	23,90	1,31
PSMC1	NM_002802	proteasome (prosome, macropain) 26S subunit, ATPase, 1	23,75	1,31
IMP3	NM_018285	IMP3, U3 small nucleolar ribonucleoprotein, homolog (yeast)	23,48	1,31
RNU3IP2	BC023662	RNA, U3 small nucleolar interacting protein 2	23,41	1,31
SMAD3	NM_005902	SMAD, mothers against DPP homolog 3 (Drosophila)	21,49	1,27
GPX2	NM_002083	glutathione peroxidase 2 (gastrointestinal)	21,27	1,27
LSP1	NM_002339	lymphocyte-specific protein 1	21,21	1,27
FGG	NM_021870	fibrinogen gamma chain	18,37	1,23
C20orf94	NM_001009608	chromosome 20 open reading frame 94	16,04	1,19
PPIL1	NM_016059	peptidylprolyl isomerase (cyclophilin)-like 1	14,74	1,17
HOXB2	NM_002145	homeobox B2	13,91	1,16
APOH	NM_000042	apolipoprotein H (beta-2-glycoprotein I)	13,17	1,15
PRSS23	NM_007173	protease, serine, 23	10,41	1,12
IARS2	NM_018060	isoleucine-tRNA synthetase 2, mitochondrial	10,02	1,11

#### Hierarchical clustering of differentially expressed genes

**Figure 3:**
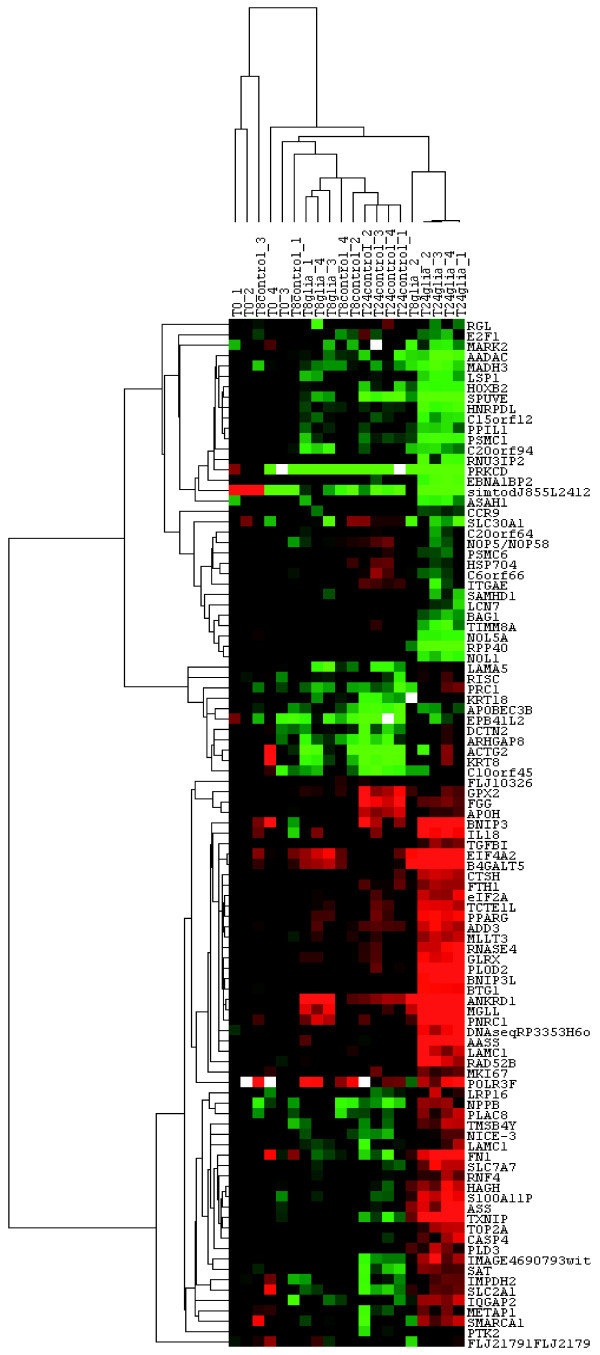
**Hierarchical clustering of the 116 identified genes expression data**. Four individual microarrays were used per condition. Hierarchical clustering was performed on conditions and on the 116 genes identified with Genespring. Each ratio was normalized to the median of the t = 0 hour-condition values of the corresponding gene. Each column represents an individual array (T0: t = 0 hour condition samples; T8control: t = 8 hours of culture without EGC; T8glia: t = 8 hours of culture in presence of EGC; T24control: t = 24 hours of culture without EGC; T24glia: t = 24 hours of culture in presence of EGC). Each line represents one individual gene. The clustering reveals clusters of genes with similar pattern of expression among the different conditions. The cluster also shows the distance between the five conditions demonstrating major changes induced by the culture with EGC at t = 24 hours.

Hierarchical clustering was used to visualize the expression profile of the 116 genes induced or repressed by EGC after 24 hours of culture (Figure 3).

All these genes exhibit a differential expression between control and EGC conditions at t = 24 hours. Furthermore, some of them already exhibited a slight difference in expression profile between control and EGC conditions at 8 hours. These results indicate that EGC effects on genes identified as differentially expressed in IEC at 24 hours probably started as early as at 8 hours, even though the modifications were not statistically significant.

Two groups of samples exhibited a very different profile from other samples: EGC condition at t = 24 hours and controls at t = 24 hours (Figure 3). These observations confirm that 1) no major changes existed between control and EGC conditions at t = 8 hours and 2) that the 24 hour-time of culture impacted on gene expression profiling in IEC, likely reflecting differentiation of IEC over the time of culture.

### EGC regulate IEC functions

#### Gene network interactions

Biological interactions among the 116 genes of the gene set provided by Genespring analysis were identified using Ingenuity Pathways Analysis. Among the 116 genes differentially expressed, Ingenuity identified 92 genes contributing to a total of 10 functional networks (Table 3). Each of the 6 first networks contained at least 14 genes that were associated with cell-to-cell signalling and interaction, cellular growth and proliferation, cell morphology, cellular movement, cell death and cell cycle. The 116 genes were also classified into Ingenuity cellular and molecular pathways as well as into Ingenuity signalling pathways (Table 4 and 5). All the functions described above and identified by building functional networks among our gene set were found in the 25 cellular and molecular functions obtained with Ingenuity (Table 4). Moreover, these 6 functions were among the 10 first functions presenting the highest score (Table 4). Finally, the signalling pathways identified by the Ingenuity analysis of our gene set were also relevant to those 6 functions (Table 5). The limit of Ingenuity analysis for our study is that it is not restricted to one specific organ or cell, so that all the results of Ingenuity analysis could not be transposed directly to the regulation of IEC functions by EGC. We therefore performed an "epithelial" specific analysis of the major functions identified with Ingenuity.

**Table 3:** Lists of differentially expressed genes involved in functional networks regulated in intestinal epithelial cells by enteric glial cells.

Ingenuity^© ^top functions	Genes list	Score
Cell-To-Cell Signaling and Interaction Cell Morphology Tissue Development	CDH1, FN1, ITGAE, KRT8, KRT18, MGLL, NPPB, PKN2, PPARG, PRKCD, PSMC1, PSMC6, PTK2, SAT, SMAD3, TGFBI, VIP	26

Cellular Growth and Proliferation Cancer Gene Expression	B4GALT5, BAG1, BNIP3 (includes EG:664), CCR9, DCTN2, EBNA1BP2, HCAP-D3, LSP1, MLLT3, PNRC1, PPIL1, PRC1, RNF4, SAT, SLC30A1, TP53RK	24

Cell Morphology Cellular Development Cardiovascular System Development and Function	AASS, ACTG2, BNIP3L, BTG1, CTSH, DYNLT3, EIF4A2, GLRX, MKI67, PLOD2, RNASE4, SLC2A1, SLC7A7, TXNIP	20

Cellular Movement Hematological System Development and Function Immune Response	APOBEC3B, ASAH1, C6, FTH1, IL18, IQGAP2, LAMA5, LAMC1, NOL5A, NOP5/NOP58, PLD3, PRKCQ, PRSS23, SCPEP1	20

Cell Death Cancer Gastrointestinal Disease	APOH, ASS, BDP1, BTG1, CARD12, CASP4, E2F1, FGFR2, FGG, NOL1, RGL1, SMARCA1, SNX2, TOP2A	20

Cell Cycle Gastrointestinal Disease Developmental Disorder	ADD3, ANKRD1, CDK5R1, EPB41L2, GPX2, HNRPDL, HOXB2, IMPDH2, MARK2, PKN2, PLAC8, POLR3F, SFRP4, TUBB3	20

RNA Post-Transcriptional Modification	IMP3	2

RNA Post-Transcriptional Modification	EIF2A	2

Protein Trafficking Cellular Compromise Auditory and Vestibular System Development and Function	TIMM8A	1

RNA Post-Transcriptional Modification RNA Damage and Repair	RPP40	1

**Table 4:** Lists of differentially expressed genes involved in cellular and molecular functions regulated in intestinal epithelial cells by enteric glial cells.

Ingenuity^© ^cellular and molecular functions	Genes list	Score
RNA Post-Transcriptional Modification	NOP5/NOP58, NOL5A, EBNA1BP2, RNU3IP2, IMP3	5.17
Cell Death	GPX2, SAT, PRKCD, B4GALT5, TXNIP, TOP2A, CDH1, PKN2, ASAH1, LSP1, BNIP3L, NPPB, CDK5R1, ANKRD1, VIP, CASP4, GLRX, FGFR2, KRT18, BAG1, PRKCQ, BTG1, PPARG, C6ORF66, SLC2A1, CARD12, SMAD3, FTH1, LAMA5, PTK2, IL18, MLLT3, FN1, PLAC8, KRT8, TGFBI, BNIP3, E2F1, SFRP4	4.85
Cell-To-Cell Signaling and Interaction	APOH, PRKCD, SMAD3, CDH1, PKN2, LAMA5, PTK2, VIP, IL18, FN1, FGG, KRT8, KRT18, BAG1, ITGAE, LAMC1, PPARG, TGFBI, E2F1	3.92
Cellular Development	SMAD3, PRKCD, CDH1, LAMA5, VIP, PTK2, IL18, CDK5R1, FGFR2, FN1, PLAC8, PRKCQ, LAMC1, PPARG, E2F1	3.92
Cell Morphology	SMAD3, PRKCD, CDH1, LAMA5, LSP1, DCTN2, VIP, IL18, PTK2, CDK5R1, FN1, KRT8, KRT18, PPARG, TGFBI, E2F1	3.88
Cellular Assembly and Organization	APOH, PRKCD, SMAD3, TOP2A, CDH1, DCTN2, EBNA1BP2, NPPB, PTK2, CDK5R1, HCAP-D3, FN1, FGG, KRT8, KRT18, MARK2, LAMC1, PPARG, BNIP3, E2F1	3.88
Carbohydrate Metabolism	FN1, B4GALT5, NPPB, PTK2, IL18	3.11
Cellular Movement	HOXB2, CCR9, C6, B4GALT5, SMAD3, PRKCD, MGLL, CDH1, LSP1, LAMA5, DCTN2, NPPB, CDK5R1, VIP, PTK2, IL18, FN1, BAG1, ITGAE, PPARG, TGFBI	3.08
Cellular Growth and Proliferation	SAT, PRKCD, TXNIP, SMAD3, CDH1, FTH1, LAMA5, SLC30A1, EBNA1BP2, VIP, IL18, PTK2, MLLT3, FGFR2, FN1, PLAC8, BAG1, PRKCQ, BTG1, LAMC1, PPARG, BNIP3, E2F1, SFRP4	3.05
Cell Cycle	HCAP-D3, FN1, TXNIP, PRKCD, TOP2A, DCTN2, PPARG, EBNA1BP2, E2F1, VIP	2.57
Molecular Transport	FGFR2, SAT, FN1, PRKCD, MGLL, BAG1, FTH1, PPARG, NPPB, PTK2, VIP, IL18	2.45
Nucleic Acid Metabolism	SAT, BAG1, NPPB, VIP	2.45
Small Molecule Biochemistry	APOH, SAT, PRKCD, B4GALT5, MGLL, FTH1, ASAH1, NPPB, PTK2, IL18, VIP, ASS, GLRX, FGFR2, FN1, BAG1, PPARG	2.45
Cellular Function and Maintenance	CCR9, SMAD3, CDH1, SLC30A1, PTK2, IL18, CDK5R1, FN1, FGG, KRT18, ITGAE, PPARG, BNIP3	2.25
DNA Replication, Recombination, and Repair	HCAP-D3, FN1, SMAD3, PRKCD, TOP2A, FTH1, DCTN2, EBNA1BP2	2.17
Gene Expression	APOH, SMAD3, PRKCD, CDH1, PKN2, VIP, IL18, FN1, BAG1, PRKCQ, RNF4, PPARG, E2F1	2.13
Cell Signaling	ASS, FN1, PRKCD, PPARG	2.02
Amino Acid Metabolism	ASS, FTH1	1.94
Cellular Compromise	PRKCD, KRT18, TIMM8A, PPARG, E2F1	1.94
Drug Metabolism	GLRX, FTH1, NPPB, IL18, VIP	1.94
Lipid Metabolism	FGFR2, APOH, SAT, FN1, MGLL, ASAH1, PPARG, NPPB, VIP	1.94
Post-Translational Modification	PRKCD, BAG1, PRKCQ	1.94
Protein Folding	BAG1	1.94
Protein Synthesis	BAG1, IL18	1.94
Vitamin and Mineral Metabolism	FGFR2, FTH1, PPARG	1.94

**Table 5:** Lists of differentially expressed genes involved in signalling pathways regulated in intestinal epithelial cells by enteric glial cells.

Ingenuity^© ^Signalling Pathway	Genes	Ratio
Circadian Rhythm Signaling	VIP	0.046
Cell Cycle: G1/S Checkpoint Regulation	SMAD3, E2F1	0.041
Integrin Signaling	ACTG2, FN1, LAMA5, LAMC1, PTK2	0.03
Actin Cytoskeleton Signaling	TMSB4Y, FGFR2, FN1, ITGAE, IQGAP2, PTK2	0.029
Cell Cycle: G2/M DNA Damage Checkpoint Regulation	TOP2A	0.029
VEGF Signaling	ACTG2, PTK2	0.029
Complement and Coagulation Cascades	FGG, C6	0.028
Amyloid Processing	CDK5R1	0.028
ERK/MAPK Signaling	PRKCD, PPARG, PTK2	0.024
Wnt/β-catenin Signaling	CDH1, MARK2, SFRP4	0.022
FGF Signaling	FGFR2	0.018
Chemokine Signaling	PTK2	0.018
TGF-β Signaling	SMAD3	0.016
Protein Ubiquitination Pathway	PSMC6, PSMC1, BAG1	0.016
PPAR Signaling	PPARG	0.015
IGF-1 Signaling	PTK2	0.015
Apoptosis Signaling	PRKCQ	0.015
Neuregulin Signaling	CDK5R1	0.015
PTEN Signaling	PTK2	0.014
Fc Epsilon RI Signaling	PRKCD	0.014
T Cell Receptor Signaling	PRKCQ	0.014
Xenobiotic Metabolism Signaling	PRKCD, PRKCQ	0.010
NF-κB Signaling	PRKCQ	0.009
B Cell Receptor Signaling	PRKCQ	0.009
Ephrin Receptor Signaling	PTK2	0.009
Leukocyte Extravasation Signaling	PTK2	0.008
Huntington's Disease Signaling	CDK5R1	0.007
Axonal Guidance Signaling	PTK2	0.004

#### Cell-to cell and cell-to-matrix interaction

EGC regulated the expression of numerous genes involved in the control of IEC adhesive processes. In particular, EGC induced an up-regulation of the expression of all 7 genes with pro-adhesive functions and a down-regulation of the 2 genes with anti adhesive properties, among the gene set found to be differentially expressed in IEC cultured in presence of EGC (Table 6). These genes are crucially involved in the control of cell-to-cell and cell-to-matrix adhesion.

**Table 6:** Genes controlling intestinal epithelial cells adhesion and modulated by enteric glial cells.

Pro-adhesive		Anti-adhesive	
**Gene Symbol**	**Regulation of gene expression by EGC**	**Gene Symbol**	**Regulation of gene expression by EGC**
CDH1	up-regulated	CDK5R1	down-regulated
IQGAP2	up-regulated	KLK14	down-regulated
LAMA5	up-regulated		
LAMC1	up-regulated		
FN1	up-regulated		
PTK2	up-regulated		
KRT8	up-regulated		

First, EGC concomitantly increased the expression of *CDH-1*, which encodes E-cadherin, and decreased the expression of *CDK5R1*. E-Cadherin is the major component of the adherent junction complexes and the level of E-Cadherin in IEC is to be correlated to adhesion complexes formation between IEC [16,17]. Further evidences confirming a pro-adhesive influence of EGC on IEC is the EGC-induced down-regulation of *CDK5R1 *expression. Indeed, *CDK5R1 *encodes p35, a regulator of CDK-5 (cyclin-dependent kinase), which induces the degradation of E-Cadherin precursor [18]. In addition, EGC also up-regulated *IQGAP2 *expression in IEC. This gene encodes for a protein member of IQGAP family that interacts with several molecules controlling cytoskeleton organization, cell adhesion and cell motility such as CDC42 and Rac [19]. Interestingly, IQGAP2 has been shown to mediate E-Cadherin-based cell-to-cell adhesion during development [20]. All these results suggest that EGC enhance cell-to-cell adhesion in IEC.

Our data also demonstrate that EGC modulate the expression of genes that are involved in cell-to-matrix interactions. First, EGC increased expression of several genes encoding proteins of the extracellular matrix such as *LAMA5*, *LAMC1 *and *FN1*. *LAMA5 *and *LAMC1 *encode respectively for laminin α5 and γ1 chains which, together with laminin β1 chain, compose laminin-10 [21]. Laminin-10 has been shown to be the most adhesive substratum of laminin isoforms when studying abilities of laminin-2,-5 and -10 in modulating Caco-2 cell adhesion [22]. Furthermore, EGC up-regulated *FN1 *expression, encoding the fibronectin protein. Interestingly, fibronectin has recently been shown to enhance Caco-2 cell attachment and wound healing [23]. EGC down-regulated *KLK14 *expression, which encodes KLK (kallikrein) 14, an extracellular serine protease which has been shown to cleave and digest various extracellular matrix proteins such as collagen IV, laminin and fibronectin [24]. In addition, EGC up-regulated *PTK2 *expression in IEC which may result in increased expression of FAK (Focal Adhesion Kinase) protein, a major regulator of focal adhesions turnover and maturation [25]. Finally, EGC induced an up-regulation of *KRT8 *expression whose increased expression has recently been shown to cause enhanced adhesion of human breast tumor cells to their extracellular matrix [26].

**Figure 4:**
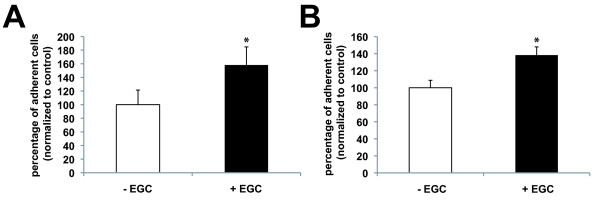
**Enteric glial cells (EGC) induced an increase in intestinal epithelial cells(IEC) adhesion**. (A):EGC induced a significant increase in IEC total adhesion (i.e. without discriminating cell-to-matrix and cell-to-cell adhesion) after 24 hours of co-culture in presence of EGC (+ EGC) as compared to control (- EGC) (n = 5; p = 0.008; Mann-Whitney test). (B): IEC were significantly more attached to their matrix when they were cultured for 24 hours with EGC (+ EGC) as compared to control (- EGC) (n = 13; p < 0.001; Mann-Whitney test).

In conclusion, our data suggest that EGC regulation of IEC transcriptome leads to an increase in cell adhesion. In order to functionally validate this hypothesis, we performed *in vitro *experiments using established adhesion assays. Under these conditions, we first showed that IEC global adhesion was increased after 24 hours of culture with EGC as compared to control (Figure 4A). We next confirmed whether these effects were in part associated with an increase in cell-to-matrix adhesion as the majority of IEC genes regulated by EGC presence appeared to favor cell-to-matrix adhesion. Indeed, cell-to-matrix adhesion assays revealed that EGC significantly increased IEC adhesion to the filter as compared to control (Figure 4B).

#### Cell differentiation

EGC also regulated the expression of numerous genes involved in IEC differentiation. In particular, EGC up-regulated the expression of 6 genes enhancing differentiation and down-regulated 3 genes known to inhibit IEC differentiation (Table 7).

**Table 7:** Genes controlling intestinal epithelial cells differentiation and modulated by enteric glial cells.

Pro-differentiative		Anti-differentiative	
**Gene Symbol**	**Regulation of gene expression by EGC**	**Gene Symbol**	**Regulation of gene expression by EGC**
CDH1	up-regulated	E2F1	down-regulated
PPARG	up-regulated	BAG1	down-regulated
LAMA5	up-regulated	CDK5R1	down-regulated
PTK2	up-regulated	FN1	up-regulated
DCTN2	up-regulated		
DYNLT3	up-regulated		

EGC induced an up-regulation of the expression of pro-differentiative genes or genes associated with enhanced differentiation of IEC such as *PPARG*, *LAMA5*, *PTK2*, *CDH-1, DCTN2 *and *DYNLT3*. Indeed, PPARγ, encoding the well-described nuclear receptor superfamily member peroxisome proliferator-activated receptor gamma (PPAR-γ) has been shown to regulate IEC differentiation and its expression has been positively correlated with level of differentiation of Caco-2 and HT29 cells [27,28]. Moreover, a diminution of laminin-a5 in a murine model resulted in a transformation from a small intestinal to a colonic mucosal architecture, suggesting that laminin-α5 has a crucial role in establishing and maintaining the architecture of the small intestine [29]. In addition, it has already been shown that the differentiation of Caco-2 cells was accompanied by an increase in FAK expression [30]. E-Cadherin, whose corresponding gene *CDH-1 *is up-regulated by EGC, has been largely demonstrated to be involved in the establishment of a differentiated phenotype for IEC. Notably, E-Cadherin has been described to be less expressed at the bottom of the crypts where IEC are undifferentiated [16,31,32]. The down-regulation by EGC of *CDK5R1 *expression, leading to enhanced levels of E-Cadherin (see previous paragraph), might also further enhance EGC-induced cell differentiation. EGC also increased *DCTN2 *and *DYNLT3 *expression, two genes encoding a subunit of dynactin (p50 or dynamitin) and dynein light chain rp3, respectively. Both are involved in post-Golgi movement of vesicles towards apical surface of differentiated enterocytes [33-35], and could therefore reflect increased differentiation of IEC induced by EGC. Intriguingly, although differentiation of the Caco-2 cell line has been shown to be correlated with a down-regulation in fibronectin expression [36], EGC induced an up-regulation in *FN1 *expression in IEC in our study.

EGC decreased the expression of genes that encode proteins implicated in anti-differentiative pathways such as *E2F1, BAG1 *and *CDK5R1 *(discussed above). *E2F1 *is a gene encoding a protein member of the E2F family of transcription factors and has been shown to be down-regulated in confluent human IEC and differentiated enterocytes [37]. *BAG1*, encoding a Bcl-2 non-homologous associated molecule, has also been shown to present a decreasing gradient of expression from the base of the crypts to the apex of the villi, suggesting that the down-regulation of *BAG1 *might reflect a differentiation state of IEC [38].

In conclusion, based on our analysis, EGC-mediated regulation of IEC transcriptome appears to strongly favor IEC differentiation.

#### Cell motility

EGC regulated in IEC the expression of genes encoding proteins that are known to play a role in IEC motility (Table 8). In particular, EGC induced an increase in *FN1 *expression in IEC. *FN1 *has been demonstrated as a major factor in promoting cell migration of IEC and subepithelial fibroblasts, thus favoring epithelial wound healing [39,40]. Interestingly, EGC induced a down-regulation in *LSP1 *expression in IEC. *LSP1 *gene encodes for LSP1, a cytoplasmic actin-binding protein, whose overexpression in melanoma cells has been described to inhibit cell motility [41]. EGC-induced up-regulation of *PTK2 *expression also supports a role of EGC in promoting IEC motility. Indeed, increased FAK protein level promoted epithelial restitution *via *an increased IEC migration [42,43]. Similarly, the increased PPARγ expression could enhance cell motility as inhibitors of PPARγ inhibit epithelial cell migration [44-46].

**Table 8:** Genes controlling intestinal epithelial cells motility and modulated by enteric glial cells.

Pro-motility		Anti-motility	
**Gene Symbol**	**Regulation of gene expression by EGC**	**Gene Symbol**	**Regulation of gene expression by EGC**
PPARG	up-regulated	LSP1	down-regulated
FN1	up-regulated		
PTK2	up-regulated		

#### Cell proliferation

Expression of genes involved in cell proliferation was differentially regulated in IEC cultured in presence of EGC as compared to control (Table 9). In fact, EGC appeared to modulate the expression of anti-proliferative and pro-proliferative genes toward a dominant anti-proliferative effect (Table 9).

**Table 9:** Genes controlling intestinal epithelial cells proliferation and modulated by enteric glial cells.

Pro-proliferative		Anti-proliferative	
**Gene Symbol**	**Regulation of gene expression by EGC**	**Gene Symbol**	**Regulation of gene expression by EGC**
E2F1	down-regulated	TXNIP	up-regulated
FGFR2	down-regulated	BTG1	up-regulated
PPIL1	down-regulated	TP53RK	down-regulated
MKI67	up-regulated	SFRP4	down-regulated

The expression of major anti-proliferative and pro-proliferative genes was found to be up-regulated and down-regulated, respectively, by EGC. In particular, *PPARG*, *TXNIP *and *BTG1 *expressions in IEC were up-regulated by EGC. PPARγ activation has been described both *in vivo *and *in vitro *to inhibit intestinal epithelial cell proliferation [47,48] and to induce a G1 phase cell cycle arrest [27]. Furthermore, *TXNIP *encodes the thioredoxin-interacting protein, a negative regulator of thioredoxin. Thioredoxin is an important growth-promoting factor of IEC [49]. Moreover, *TXNIP *has also recently been suggested to be a tumor suppressor gene in hepatocellular carcinoma [50] and interestingly, *TXNIP *expression is decreased in colorectal cancer and ulcerative colitis [51]. Similarly, *BTG1 *has been shown to negatively regulate cell proliferation and to present a maximal expression during G0/G1 phases of the cell cycle in fibroblasts [52]. Further reinforcing the anti-proliferative effects of EGC on IEC is the EGC-induced down-regulation of the expression of pro-proliferative genes such as *E2F1*, *FGFR2 *and *PPIL1. E2F1 *is a gene encoding a protein member of the E2F family of transcription factors that regulate cell cycle progression by modulating expression of proteins required for the G1/S transition. It has been well described that growth stimulatory signals lead to active E2F1 accumulation and S-phase entry [53,54]. *FGFR2 *encodes a member of the FGF (Fibroblast Growth Factor) receptor family with high affinity for KGF (Keratinocyte Growth Factor) which is a major actor in the mesenchymal stimulation of epithelial cell proliferation [55,56]. Finally, *PPIL1*, which encodes a cyclophilin-related protein, PPIL1 (peptidyl prolyl isomerase-like protein), implicated in spliceosome activation, has recently been described to be overexpressed in colon tumors and *PPIL1 *silencing led to an inhibition of colon cancer cell growth [57,58].

These global anti-proliferative effects of EGC upon IEC have to be associated with the EGC-induced modulation of genes that would tend to be pro-proliferative, although these are clearly in reduced numbers. For instance, EGC increase *MKI67 *expression, which encodes the proliferation marker Ki-67. Indeed, Ki-67 is increasingly expressed during the cell cycle phases [59], excepted in G0 or in cells just escaping from G0 [60]. Its function is still unclear but knock-down for Ki-67 in cancerous cells leads to an inhibition of proliferation mainly *via *an induction of apoptosis [61,62]. Interestingly, EGC reduced the expression of *TP53RK *and *SFRP4 *in IEC that encode proteins involved in anti-proliferative pathways. *TP53RK *encodes PRPK which is a short kinase that phosphorylates p53, enhancing its transcriptional activity [63] and suppressing cell cycle transition G1/S [64]. *SFRP4 *encodes the protein sFRP4 (secreted frizzled-related protein), which is an inhibitor of the Wnt-signaling cascade through binding and sequestering Wnt ligand and, thus, has been shown to decrease cell proliferation in many cell lines [65-67].

Taken together, these data suggest that EGC tend to shift IEC transcriptome toward an anti-proliferative phenotype. These results could lead to the identification of specific targets responsible for the anti proliferative effects of EGC previously reported [12]. In addition, this global effect is supported further by the observation that EGC inhibit cell proliferation in part by inducing a cell cycle arrest in G0/G1 phase [11].

#### Cell survival

EGC differentially regulated in IEC the expression of genes involved in cell death. EGC appeared to modulate the expression of anti-apoptotic and pro-apoptotic genes toward a dominant pro-apoptotic effect (Table 10).

**Table 10:** Genes controlling intestinal epithelial cells survival and modulated by enteric glial cells.

Pro-apoptotic		Anti-apoptotic	
**Gene Symbol**	**Regulation of gene expression by EGC**	**Gene Symbol**	**Regulation of gene expression by EGC**
BNIP3	up-regulated	BAG1	down-regulated
CASP4	up-regulated	ASAH1	down-regulated
CARD12	down-regulated	GPX2	down-regulated
		TUBB3	up-regulated

Indeed, expressions of pro-apoptotic and anti-apoptotic genes were found to be up-regulated and down-regulated, respectively, by EGC. In particular, *BNIP3 *and *CASP4 *expression in IEC were up-regulated by EGC. *CASP4*, coding for the caspase-4 pro-apoptotic protein has been shown to induce cell death [68,69], like *BNIP3 *which encodes a pro-apoptotic protein member of Bcl-2 family [70,71]. Conversely, *ASAH-1, GPX2 *and *BAG-1 *were down-regulated by EGC. *BAG-1 *encodes a known anti-apoptotic protein implicated in Bcl-2 signalling pathway [72,73]. *ASAH-1 *encodes acid ceramidase, an enzyme that catabolizes ceramide into sphingosine by deacylation. Overexpression of acid ceramidase in cells confers on them an increased resistance to cell death induced by various factors such as TNF (tumor necrosis factor) or anti-cancerous drugs [74,75]. Finally, *GPX2 *encodes a member of the glutathione peroxidase (GPX) family and is a selenoprotein and a glutathione peroxidase. GPX2 is expressed in IEC [76] and inhibits oxidative stress-induced apoptosis in the human breast adenocarcinoma cell line MCF-7 [77].

These global pro-apoptotic effects of EGC upon IEC have to be considered also in view of the EGC-mediated regulation of genes which would tend to be anti-apoptotic, although these are in reduced number. In particular, EGC up-regulated the expression of *TUBB3*, a gene encoding the class III isotype of β-tubulin. Silencing of class III β-tubulin by siRNA reverted anti-cancer agent-resistant cells to a sensitive phenotype and promoted apoptosis [78,79]. Conversely, EGC inhibited the expression of *CARD12 *which encodes the CARD12 protein, a member of the CED4/Apaf-1 family and known to induce apoptosis when expressed in cells [80,81].

EGC-induced regulation of genes involved in cell death has probably no clear consequences on IEC survival. This is consistent with a previous study showing that EGC did not modify IEC survival [12].

## Conclusion

The present study described the impact of EGC upon the transcriptome of proliferating Caco-2 cells in a validated non-contact co-culture model of EGC and IEC [12,13]. The results obtained confirmed the known role of EGC in the control of some IEB functions and, more interestingly, extended their role in the control of novel major IEB and IEC functions. This study further reinforced the emerging concept that EGC are an important component of the IEB environment with major protective effects. Indeed, the major pathways regulated by EGC in IEC identified with microarrays lead to enhanced cell adhesion, differentiation, and motility, which could favor repair, and reduced cell proliferation.

An important result of this study is the putative identification of genes involved in the anti-proliferative effects of EGC. Indeed, EGC have been shown to have potent anti-proliferative effects upon IEC [11,12]. Interestingly, these effects were associated with an induction of a cell cycle blockade in the G0/G1 phase [11] but were not associated with significant cell death [12]. These results are globally confirmed, as there was no clear trend in the EGC-induced modulation of genes controlling cell survival in IEC but a trend toward an up-regulation of the expression of genes involved in anti-proliferative pathways.

A major finding of our study is that EGC regulated the expression of genes involved in cell adhesion and differentiation toward a global increase of IEC adhesive properties. These results can be analyzed in view of the known effects of EGC upon IEB. Indeed, *in vitro *studies have shown that EGC increase IEB resistance and decrease IEB paracellular permeability [13]. In the present study, we also demonstrated that EGC could increase global IEB adhesion, in part by increasing cell-to-matrix adhesion. These results are in agreement with *in vivo *data showing that selective lesions of EGC lead to increased paracellular permeability and major IEB breakdown associated with the development of intestinal inflammation. However, the role of the molecular actors involved in these processes such as fibronectin, laminin or cytokeratin remains to be investigated. EGC might also favor barrier integrity by increasing its resistance to inflammatory stress either by its ability to down-regulate inflammatory genes such as *CARD12 *or by increasing IEC production of anti-inflammatory mediators such as VIP [82,83].

Another important finding of this study is the observation that EGC might regulate IEC metabolism. In particular, EGC up-regulated genes involved in lipid metabolism such as *AADAC*, *MGLL *or *APOH*, encoding respectively the arylacetamide deacetylase, monoglyceride lipase (MGL) and Apolipoprotein H [84-86]. Interestingly, inhibitors of MGL which is a serine hydrolase that converts 2-arachidonoylglycerol, a ligand of canabinoid receptors, to fatty acids and glycerol, increased gut transit time [87] but its impact on IEB functions remain unknown. EGC also modulated the expression of genes involved in protein metabolism such as *CTSH *that encodes cathepsin H, a lysosomal cysteine proteinase [88]. In addition, EGC increased the expression of genes involved in arginine metabolic pathway that are *SLC7A7 *and *ASS*, which encode respectively for the cationic amino acid transporter y(+)LAT1 and the argininosuccinate synthetase, enzyme catalyzing the penultimate step of the arginine biosynthetic pathways. The functional impact of EGC upon IEC metabolism needs to be investigated in future studies.

Regulation of IEB functions by EGC occurs mainly *via *paracrine pathways. The majority of EGC effects upon IEB functions are reproduced by glial-derived conditioned medium. In addition, various mediators have been identified as being involved in the control of cell proliferation or paracellular permeability. Our study supports the role of mediators such as TGF-β1 as a regulator of gene pathways modulated by EGC in IEC. In fact, TGF-β1 has been shown to increase the expression of FAK [43], TGFBI [89] or VIP [90]. EGC have also been shown to produce IL-6 [91]. IL-6 has recently been identified as a key molecule involved in IEB barrier protection *via *increasing both cytokeratin 8 and cytokeratin 18 proteins expression [92], whose mRNA expression were induced by EGC in IEC in our study. In this context, knowledge of genes modulated by EGC could direct future efforts aimed at identifying novel glial mediators involved in EGC control of IEB functions. Our data also further suggest that EGC differentially regulate some IEB functions as compared to fibroblasts, although comparison has only been performed on a limited set of genes and one cannot fully rule out that species differences could also be involved (fibroblasts of human origin *vs. *enteric glia of rat origin). However, these differences are consistent with the observation that while EGC have anti-proliferative effects on both human and rat IEC [12], fibroblasts increase IEC proliferation [93].

Collectively, our data support the concept that EGC play a key protective role upon IEB homeostasis by reinforcing global barrier functions. Additionally, our study reinforces data suggesting that enteric glia lesions and/or functional defects could be involved in the development of pathologies with altered barrier (such as inflammatory bowel diseases or colorectal cancer) and also be associated with increased barrier susceptibility to pathogen aggression.

## Methods

### Cell culture

CRL2690 (ATCC), a transformed EGC line isolated from adult rat myenteric plexus, was cultured in DMEM (4.5 g/L glucose; Gibco) supplemented with 10% heat-inactivated FBS, 2 mM glutamine (Gibco), 50 IU/mL penicillin and 50 μg/mL streptomycin. EGC were seeded at a concentration of 30,000 cells/mL in 6- and 12-well plates (Corning, Avon, France). EGC were cultured for an additional 24 hours after having reached confluence prior co-culture with IEC. CCD-18Co, a human colonic fibroblast cell line, was cultured in MEM Alpha Medium (Gibco) supplemented with 10% heat-inactivated FBS, 2 mM glutamine (Gibco), 0.1 mM MEM NEAA (Gibco), 50 IU/mL penicillin and 50 μg/mL streptomycin. Fibroblasts were seeded at a concentration of 130,000 cells/mL in 12-well plates (Corning). Fibroblats were cultured in EGC medium, as described above, for an additional 24 hours after having reached confluence prior co-culture with IEC. Caco-2 cells (ATCC), isolated from a human colonic adenocarcinoma, were cultured in DMEM (4.5 g/L glucose; Gibco) supplemented with 10% heat-inactivated FBS, 2 mM glutamine (Gibco), 50 IU/mL penicillin and 50 μg/mL streptomycin and were seeded at a concentration of 140,000 cells/mL onto porous Transwell filters (6-well and 12-well Transwell clear, 0.40 μm porosity, Corning). Caco-2 cells were processed for experiment 1 day after their seeding. To characterize EGC impact onto IEC transcriptome and functions, IEC seeded onto filters were cultured in presence of EGC seeded at the bottom of the 6-well or 12-well plates.

### Microarray experiments

Transcriptomic analysis was performed with a microarray of 6,864 genes called "Cancerochip" and available from the West Genopole transcriptome core facility of Nantes. These Cancerochips contained 6,864 probes (50-mers oligonucleotides), each specific of a single gene. These genes were selected to be preferentially and/or differentially expressed in Caco-2 cells and in various tumours. Three replicates of each probe were spotted onto Cancerochips. This allowed the measurement of the probes reproducibility within the array.

Total RNA was extracted from Caco-2 cells cultured on 6-well filters alone or in presence of EGC at t = 0, 8 and 24 hours. Each condition was performed in 4 replicates allowing the measurement of the reproducibility of the cell culture experiments. RNA extraction was performed with RNeasy mini kit (Qiagen) according to the manufacturer's recommendations.

The protocols used for subsequent amplification and labelling were described in the DNA chips platform protocols. Each individual sample was compared to a reference pool consisting of Caco-2 cells transcripts of the four replicates extracted at t = 0 hour. Total RNA (0.5 μg) was amplified and labelled using the Amino Allyl MessageAmp II aRNA Amplification kit (Ambion) and CyDye Post Labelling Reactive Dyes (Amersham). After reverse transcription to synthesize first strand cDNA, second strand cDNA was subsequently synthesized following the manufacturer's protocol. *In vitro *transcription was then achieved in order to amplify the initial transcripts quantity, concomitantly with aminoallyl-dUTP incorporation to perform labelling with cyanins (Cy5 for the reference and Cy3 for samples). The hybridization of the chips was performed following the protocol of the West Genopole transcriptome core facility of Nantes. After washing, the chips were scanned (Scanexpress- Perkin Elmer).

### Data analysis

After acquisition, the scanned images were analyzed using GenePix Pro v5.1 software (Axon). Raw signals were processed using the MADSCAN package http://cardioserve.nantes.inserm.fr/mad/madscan/. Spots with weak, saturated signal or badly shaped were considered as missing values. Print-tip lowess was applied to raw signals to normalize both channels (Cy3 and Cy5) of a same array. Fitting coefficients were calculated on rank invariant spots, assuming that they correspond to ubiquitous genes (genes that did not vary between samples). Sample to reference ratios (Cy3/Cy5) were further calculated, and Log transformed (Expression Logratios). Probes with more than 25% of missing values were rejected.

In order to identify differentially expressed genes, Expression Logratios were analyzed using Genespring v7.0 software (Agilent Technologies). Genes differentially expressed between Caco-2 cells cultured alone or in presence of EGC were searched with Benjamini and Hochberg False Discovery Rate method with a significance threshold of 0.05. This method includes a correction for multi-testing and has been widely used for microarray data [94]. This analysis led to the identification of 98 genes differentially expressed in IEC cultured in presence of EGC as compared to control, i.e. IEC cultured alone at t = 24 hours and none at t = 8 hours. Analysis of variance (ANOVA) using time of culture and presence/absence of ECG as parameters gave very similar results. Data visualization using hierarchical clustering and Volcano-Plot suggested that this strategy might have missed some differentially expressed genes at t = 24 hours; we thus selected an additional set of genes based on expression fold-changes. Twenty seven genes with a fold-change threshold of 2 and a t-test p-value < 0.05 without multi-testing correction were found. Altogether 116 unique genes were found differentially expressed in IEC at t = 24 hours of culture in presence of EGC as compared to control.

Hierarchical clustering was performed after normalization on medians of the ratio values of the t = 0 hour-condition samples. Hierarchical clustering was performed using the Cluster software. It was applied to order either genes and samples or genes only. It creates a visualization of the grouping of genes and samples based on profile similarity, even if it does not provide robustness assessment of the classification.

Among the 116 genes identified with Genespring analysis, 17 of them did not present reliable values at t = 0 hour. Thus, these 17 genes were excluded from hierarchical clustering analyses. As a consequence, clustering analyses only involved 99 genes.

Microarray data were uploaded to GEO database http://www.ncbi.nlm.nih.gov/geo/ and are available under the access number GSE17027.

### RT-quantitative PCR

Extraction of total RNA from Caco-2 cells cultured alone, in presence of EGC or fibroblasts for 24 hours was performed with RNeasy Mini kit (Qiagen) according to the manufacturer's protocol. For reverse transcription, 1 μg of purified total RNA was denatured and subsequently processed for reverse transcription using SuperScript II Reverse Transcriptase (Invitrogen) according to the manufacturer's recommendations. PCR amplifications were performed using the Absolute Blue SYBR green fluorescein kit (ABgene) according to the manufacturer's protocol and run on MyiQ thermocycler (Biorad). The expression of the gene *S6 *was analyzed in parallel as an internal control.

***CDH1 ***[GenBank: NM_004360]

Forward primer:

5'-GACCAGGACTATGACTACTTGAACG-3'

Reverse primer:

5'-ATCTGCAAGGTGCTGGGTGAACCTT-3'

***E2F1 ***[GenBank: NM 005225]

Forward primer:

5'-CCGCTCGAGGAGAAGTCACGCTATGA-3'

Reverse primer:

5'-CCCAAGCTTTTGGTGATGTCATAGATGC-3'

***FN1 ***[GenBank: NM_054034]

Forward primer:

5'-GCAGGCTCAGCAAATGGTTCAG-3'

Reverse primer:

5'-AGGTAGGTCCGCTCCCACTG-3'

***FGFR2 ***[GenBank: NM_022970]

Forward primer:

5'-GTCCTGCCAAAACAGCAAG-3'

Reverse primer:

5'-CCCCTATGCAGTAAATGGCTA-3'

***GPX2 ***[GenBank: NM_002083]

Forward primer:

5'-gtccttggcttcccttgc-3'

Reverse primer:

5'-tgttcaggatctcctcattctg-3'

***LAMA5 ***[GenBank: NM_005560]

Forward primer:

5'-CCCACCGAGGACCTTTACTGC-3'

Reverse primer:

5'-GGTGTGCCTTGTTGCTGTTGG-3'

***PPARG ***[GenBank: NM_138712/NM_005037/NM_138711/NM_015869]

Forward primer:

5'-ttgctgtcattattctcagtgga-3'

Reverse primer:

5'-gaggactcagggtggttcag-3'

***PTK2 ***[GenBank: NM_153831/NM_005607]

Forward primer:

5'- GAGATCCTGTCTCCAGTCTAC-3'

Reverse primer:

5'- TGCACCTGCTATTTTTAGTTG-3'

***SMAD3 ***[GenBank: NM 005902]

Forward primer:

5'-CCAAGCTTAGAACGGGCAGGAGGAG-3'

Reverse primer:

5'-CACTCGAGTGGTGGCTGTGCAGGTC-3'

***S6 ***[GenBank: NM_001010]

Forward primer:

5'-TGGCAAGATGATCCCAATGA-3'

Reverse primer:

5'-AGCTTCTTTGGGACACCTGCT-3'

### Adhesion experiments

#### Global adhesion assay

IEC adhesion was estimated by performing a "global adhesion assay" that evaluated total IEC adhesion to their environment, i.e. adhesion to neighboring IEC and adhesion to matrix. IEC were cultivated on filters (12-well Transwell clear, 0.40 μm porosity, Corning) alone or in the presence of EGC for 24 hours. IEC were then trypsinized with 0.01% trypsin-EDTA free (Sigma) allowing gentle trypsinization for 30 minutes at 37°C. Non-adherent IEC were harvested and counted in a blind fashion using Malassez slides (VWR international). IEC remaining adhered on filters were trypsinized with 2.5% trypsin with EDTA (Gibco), harvested and counted. Results are expressed in percentage of remaining adherent IEC normalized to the total number of counted IEC (i.e., adherent IEC and non-adherent IEC). Only those series in which the percentage of IEC total adhesion in control condition was comprised between 20 and 70% were analyzed.

#### Cell-to-matrix adhesion assay

IEC were cultivated on filters (12-well Transwell clear, 0.40 μm porosity, Corning) alone or in presence of EGC for 24 hours. IEC were then trypsinized for 10 minutes with a 2.5% trypsin-EDTA (Gibco). Trypsin was neutralized with IEC culture medium (see above). IEC were subsequently reseeded on filters and incubated for 3 hours at 37°C. Time of incubation has been defined to allow 50% of seeded IEC to adhere to filters in control condition. Following incubation, unseeded cells were harvested and counted in a blind fashion using Malassez slides (VWR international). IEC that had adhered on filters were trypsinized and counted. Results are expressed in percentage of adherent IEC normalized to the total number of counted IEC (i.e., adherent IEC and non-adherent IEC). Only those series in which the percentage of IEC total adhesion in control condition was comprised between 20 and 70% were analyzed.

## List of abbreviations

**CNS**: Central nervous system; **EGC**: Enteric glial cells; **ENS**: Enteric nervous system; **FAK**: Focal adhesion kinase; **IEB**: Intestinal epithelial barrier; **IEC**: Intestinal epithelial cells; **PPARγ**: Peroxisome proliferator-activated receptor gamma; **TGF-β1**: Transforming growth factor beta-1; **VIP**: Vasoactive intestinal peptide.

## Authors' contributions

LVL designed microarrays studies and functional experiments and carried out microarrays studies. She analyzed the microarrays data and wrote the manuscript. MMM designed and carried out functional experiments and performed RT-qPCR studies. JL supervised microarray studies. IG participated in microarrays hybridization. RT assisted with the bioinformatics analysis. RH contributed to the bioinformatics analysis and wrote the manuscript. MN supervised the project and wrote the manuscript. All authors read and approved the final manuscript.
